# Predicting state transitions in the transcriptome and metabolome using a linear dynamical system model

**DOI:** 10.1186/1471-2105-8-343

**Published:** 2007-09-18

**Authors:** Ryoko Morioka, Shigehiko Kanaya, Masami Y Hirai, Mitsuru Yano, Naotake Ogasawara, Kazuki Saito

**Affiliations:** 1 RIKEN Plant Science Center, Yokohama, Kanagawa, Japan; 2 Department of Bioinformatics and Genomics, Graduate School of Information Science, Nara Institute of Science and Technology, Ikoma, Nara, Japan; 3 Department of Molecular Biology and Biotechnology, Graduate School of Pharmaceutical Science, Chiba University, Inage-ku, Chiba, Japan

## Abstract

**Background:**

Modelling of time series data should not be an approximation of input data profiles, but rather be able to detect and evaluate dynamical changes in the time series data. Objective criteria that can be used to evaluate dynamical changes in data are therefore important to filter experimental noise and to enable extraction of unexpected, biologically important information.

**Results:**

Here we demonstrate the effectiveness of a Markov model, named the Linear Dynamical System, to simulate the dynamics of a transcript or metabolite time series, and propose a probabilistic index that enables detection of time-sensitive changes. This method was applied to time series datasets from *Bacillus subtilis *and *Arabidopsis thaliana *grown under stress conditions; in the former, only gene expression was studied, whereas in the latter, both gene expression and metabolite accumulation. Our method not only identified well-known changes in gene expression and metabolite accumulation, but also detected novel changes that are likely to be responsible for each stress response condition.

**Conclusion:**

This general approach can be applied to any time-series data profile from which one wishes to identify elements responsible for state transitions, such as rapid environmental adaptation by an organism.

## Background

Biochemical systems in living cells are robust and flexible. Investigating the responses of cells (and organisms) to environmental changes typically requires a system-level analysis of the interactions between the various molecular elements (genes, enzymes, and metabolites) that comprise the system. A key step to analyze system responses to environmental changes is identifying large state changes or "transitions". A statistical method that could detect such transitions would be a powerful analytical tool for finding important factors in large-scale profiles, such as variations in gene expression.

Previous analyses of gene expression profiles have often made use of graphical models, such as Bayesian Networks [1,2], Graphical Gaussian Modelling [3], Boolean Networks [4,5], and Auto-Regressive models [6]. However, not many approaches have explicitly modelled observational noise. In Auto-Regressive analyses, for example, observational vectors **y**_*t *_are recursively defined by the following Equation [6]:

**y**_*t *_= *A***y**_*t*-1 _+ *ε*_*t*_

where ***y***_*t *_is an observational vector of genes or metabolites at time *t*, *A *is an observational transition matrix, and *ε*_*t *_is Gaussian noise. Because this model does not distinguish observational and inherent (e.g. biological) noises, identification of transition states becomes difficult in the presence of substantial noise.

Here we propose an extension of the Auto-Regressive model [6], which has been modified by the addition of reduced set of internal states, as explained in the Results and Discussion section. We chose a mathematical model, the Linear Dynamical System (LDS), as the basis of our method because it does not impose any specific requirements on the data used. LDS is expected to eliminate the confounding influence of observational noise in time series data. The model was applied to detect cellular state transitions in transcriptome and metabolome time series datasets from *Bacillus subtilis *and *Arabidopsis thaliana *maintained under stress conditions.

## Results and discussion

### Overview of our method

Our method has two steps. First, transition time points for each time series are detected using LDS, which mathematically distinguishes transitional fluctuations from experimental noise. The transition point is detected by the logarithm of the likelihood values. Here "likelihood value" means the generative probability of current data based on the condition of the past datasets. If this value is low, then the current data cannot be adequately explained by past datasets. In other words, a transition has occurred. In the second step, relevant factors such as genes and/or metabolites related to the transitions are extracted by Batch-Learning Self Organizing Mapping (BL-SOM) using changes in expression levels [7]. In summary, the LDS uses compressed information called "internal states", defined as the degenerate parameters of gene expression/metabolite accumulation profiles, to detect transitions, and then BL-SOM generates a 'Feature map', which is a two-dimensional lattice reflecting the similarity among clusters, based on the gene expression/metabolite accumulation profiles in order to visually characterize each state.

### Linear Dynamical System (LDS) for time series analyses

LDS uses internal state variables in the generative model for cellular internal state changes. These internal states correspond to the compressed description of the observed biological system prior to adding noise factors.

The total experimental dataset of the time series and the corresponding internal state are denoted by *Y*_1:*T *_= {**y**_1_, **y**_2_,..., **y**_*T*_} and *X*_1:*T *_= {**x**_1_, **x**_2_,..., **x**_*T*_}, respectively. Each element in these vectors is defined as:

**y**_*t *_= (**y**_*t*1_, **y**_*t*2_, ⋯, **y**_*tD*_)' ∈ *R*^*D*^

**x**_*t *_= (**x**_*t*1_, **x**_*t*2_, ⋯, **x**_*tN*_)' ∈ *R*^*N*^

where *t *= 1, 2,..., T is the measurement order of the time series, *D *is the dimension of vector **y**_*t *_representing expression levels of *D *genes or metabolites, and *N *is the dimension of vector **x**_*t *_representing internal states. To distinguish observational noise from true information on cellular transitions, we focus on two probability densities: the density between internal state variables *p*(**x**_*t*_|**x**_*t*-1_), and the density for evaluation of observational noise *p*(**y**_*t*_|**x**_*t*_). The proposed model is further defined as follows:

Observational equation: **y**_*t *_= *V***x**_*t *_+ ***η***_*t*_

Transition equation: **x**_*t *_= *W***x**_*t*-1 _+ ***ε***_*t*_

where *V *is a *D *× *N *observational matrix, *W *is an *N *× *N *internal state transition matrix, *D*-dimensional vector ***η***_*t *_is observational noise, *N*-dimensional vector ***ε***_*t *_is transition noise. The vectors **x**_1_, ***ε***_*t*_, ***η***_*t *_are generated according to the following equations:

**x**_1 _~ *N*_*N*_(**x**_1_|*μ*_1_, *σ*_1_^2^*I*_*N*_)

***ε***_*t *_~ *N*_*N*_(***ε***_*t*_|0_*N*_, *σ*_*ε*_^2^*I*_*N*_)

***η***_*t *_~ *N*_*D*_(***η***_*t*_|0_*D*_, *σ*_*η*_^2^*I*_*η*_)

The next step is to define the relevant probability densities. *N*_*p*_(**x**|**m**, Σ) is a probability density function when a *p*-dimensional probabilistic vector **x **obeys a Gaussian distribution whose mean vector is **m**, and covariance matrix Σ (Equation 9).

Np(x|m,∑)≡(2π)−p/2|∑|−1/2exp⁡[−12(x−m)'Σ(x−m)]
 MathType@MTEF@5@5@+=feaafiart1ev1aaatCvAUfKttLearuWrP9MDH5MBPbIqV92AaeXatLxBI9gBaebbnrfifHhDYfgasaacH8akY=wiFfYdH8Gipec8Eeeu0xXdbba9frFj0=OqFfea0dXdd9vqai=hGuQ8kuc9pgc9s8qqaq=dirpe0xb9q8qiLsFr0=vr0=vr0dc8meaabaqaciaacaGaaeqabaqabeGadaaakeaacqWGobGtdaWgaaWcbaGaemiCaahabeaakiabcIcaOiabhIha4naaeeaabaGaeCyBa0MaeiilaWIaeyyeIuUaeiykaKcacaGLhWoacqGHHjIUcqGGOaakcqaIYaGmiiGacqWFapaCcqGGPaqkdaahaaWcbeqaaiabgkHiTiabdchaWjabc+caViabikdaYaaakmaaemaabaGaeyyeIuoacaGLhWUaayjcSdWaaWbaaSqabeaacqGHsislcqaIXaqmcqGGVaWlcqaIYaGmaaGccyGGLbqzcqGG4baEcqGGWbaCcqGGBbWwcqGHsisldaWcaaqaaiabigdaXaqaaiabikdaYaaacqGGOaakcqWH4baEcqGHsislcqWHTbqBcqGGPaqkcqGGNaWjcqqHJoWucqGGOaakcqWH4baEcqGHsislcqWHTbqBcqGGPaqkcqGGDbqxaaa@6259@

We assume that the observational and internal transition noises are both Gaussian, and therefore the relationship is a first-order Markov process (Equation 10).

*p*(**x**_*t*_, **y**_*t *_| *X*_1:*t*-1_, *Y*_1:*t*-1_) = *p*(**y**_*t*_|**x**_*t*_)*p*(**x**_*t*_|*x*_*t*-1_)

The model parameter of (4)–(8) is defined as the parameter set *θ*:

*θ *= {*μ*_1_, *σ*_1_, **W**, *σ*_*ε*_, **V**, *σ*_*η*_}

Note that the model corresponds to a Kalman Filter when *θ *is known (see also Methods section) [8].

The initial state **x**_1 _is defined as:

*p*(**x**_1_|*θ*) = *N*_*N*_(**x**_1_|***μ***_1_, *σ*_1_^2^*I*_*N*_)

From Equations (5) and (7), the following function is obtained:

*p*(**x**_*t*_|**x**_*t*-1_, *θ*) = *N*_*N*_(**x**_*t*_|**Wx**_*t*-1_, *σ*_*ε*_^2^*I*_*N*_)

From Equations (4) and (8), the following function is obtained:

*p*(**y**_*t*_|**x**_*t*_, *θ*) = *N*_*D*_(**y**_*t*_|**Vx**_*t*_, *σ*_*η*_^2^*I*_*D*_)

Using these results, the following joint probability is obtained:

p(Y1:T,X1:T|θ)=p(x1|θ){∏t=2Tp(xt|xt−1,θ)}{∏t=1Tp(yt|xt,θ)}
 MathType@MTEF@5@5@+=feaafiart1ev1aaatCvAUfKttLearuWrP9MDH5MBPbIqV92AaeXatLxBI9gBaebbnrfifHhDYfgasaacH8akY=wiFfYdH8Gipec8Eeeu0xXdbba9frFj0=OqFfea0dXdd9vqai=hGuQ8kuc9pgc9s8qqaq=dirpe0xb9q8qiLsFr0=vr0=vr0dc8meaabaqaciaacaGaaeqabaqabeGadaaakeaacqWGWbaCcqGGOaakcqWGzbqwdaWgaaWcbaGaeGymaeJaeiOoaOJaemivaqfabeaakiabcYcaSiabdIfaynaaBaaaleaacqaIXaqmcqGG6aGocqWGubavaeqaaOWaaqqaaeaaiiGacqWF4oqCaiaawEa7aiabcMcaPiabg2da9iabdchaWjabcIcaOiabhIha4naaBaaaleaacqaIXaqmaeqaaOWaaqqaaeaacqWF4oqCaiaawEa7aiabcMcaPmaacmaabaWaaybCaeqaleaacqWG0baDcqGH9aqpcqaIYaGmaeaacqWGubava0qaaiabg+GivdaakiabdchaWjabcIcaOiabhIha4naaBaaaleaacqWG0baDaeqaaOWaaqqaaeaacqWH4baEdaWgaaWcbaGaemiDaqNaeyOeI0IaeGymaedabeaakiabcYcaSiab=H7aXbGaay5bSdGaeiykaKcacaGL7bGaayzFaaWaaiWaaeaadaGfWbqabSqaaiabdsha0jabg2da9iabigdaXaqaaiabdsfaubqdbaGaey4dIunaaOGaemiCaaNaeiikaGIaeCyEaK3aaSbaaSqaaiabdsha0bqabaGcdaabbaqaaiabhIha4naaBaaaleaacqWG0baDaeqaaOGaeiilaWIae8hUdehacaGLhWoacqGGPaqkaiaawUhacaGL9baaaaa@7622@

The parameter optimization follows a standard EM algorithm (see Methods section).

### Criterion for detecting internal state transitions

Using the resulting estimated parameter, the log-likelihood with respect to the present time point *t *when all time points are given is defined by Equation (16):

log *L*_*t *_= log *p*(**y**_*t*_|*Y*_1:*t*-1_, *θ*)

This is calculated using the E-step formula (see Equation 23 in Methods) after parameter estimation using the Kalman filter.

When the log-likelihood value log *L*_*t *_becomes much lower than log *L*_*t*-1_, then *y*_*t *_cannot be explained by *Y*_1:*t*-1_, i.e., the cellular internal state has changed at time *t*. In this study, the point at which the log-likelihood value becomes relatively low between whole time points is defined as the state transition point. If the log-likelihood value remains low over a certain period, then the cells are changing their states continuously during that period.

### Analysis of the *Bacillus subtilis *data

**Figure 1:**
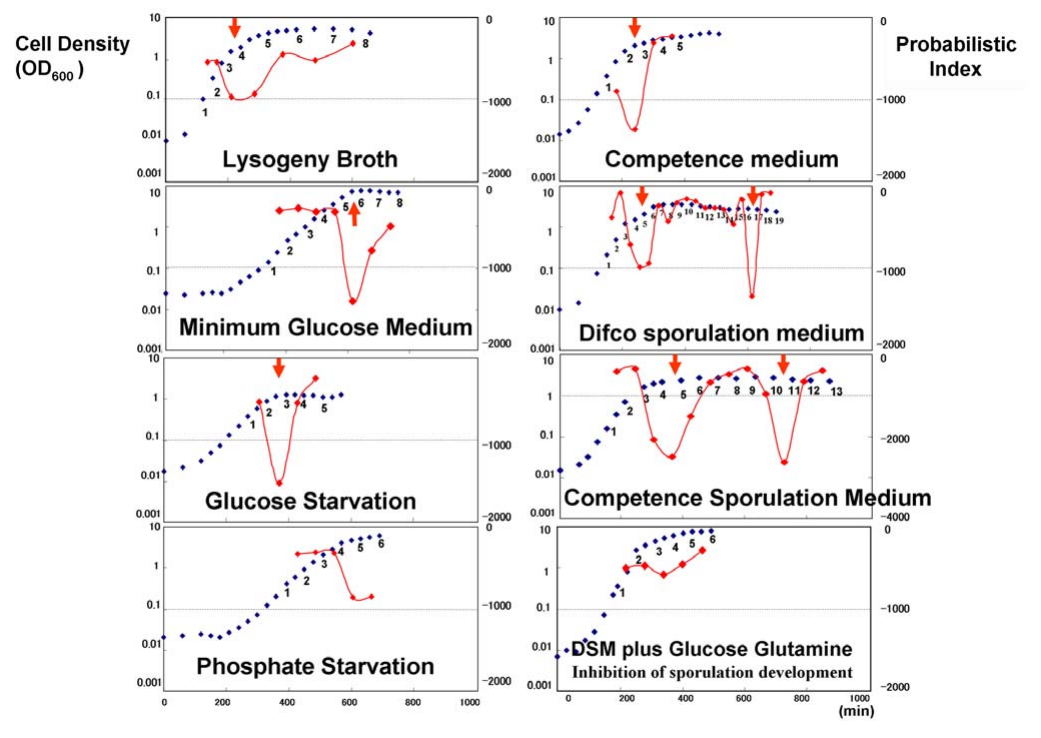
**The results of log-likelihood values and experimental conditions**. Relationship between optical density values and state transition time. Red plots show the probabilistic index for the evaluation of the state change. Blue plots show the Optical Density (OD) values that represent the cellular populations at each time.

**Figure 2:**
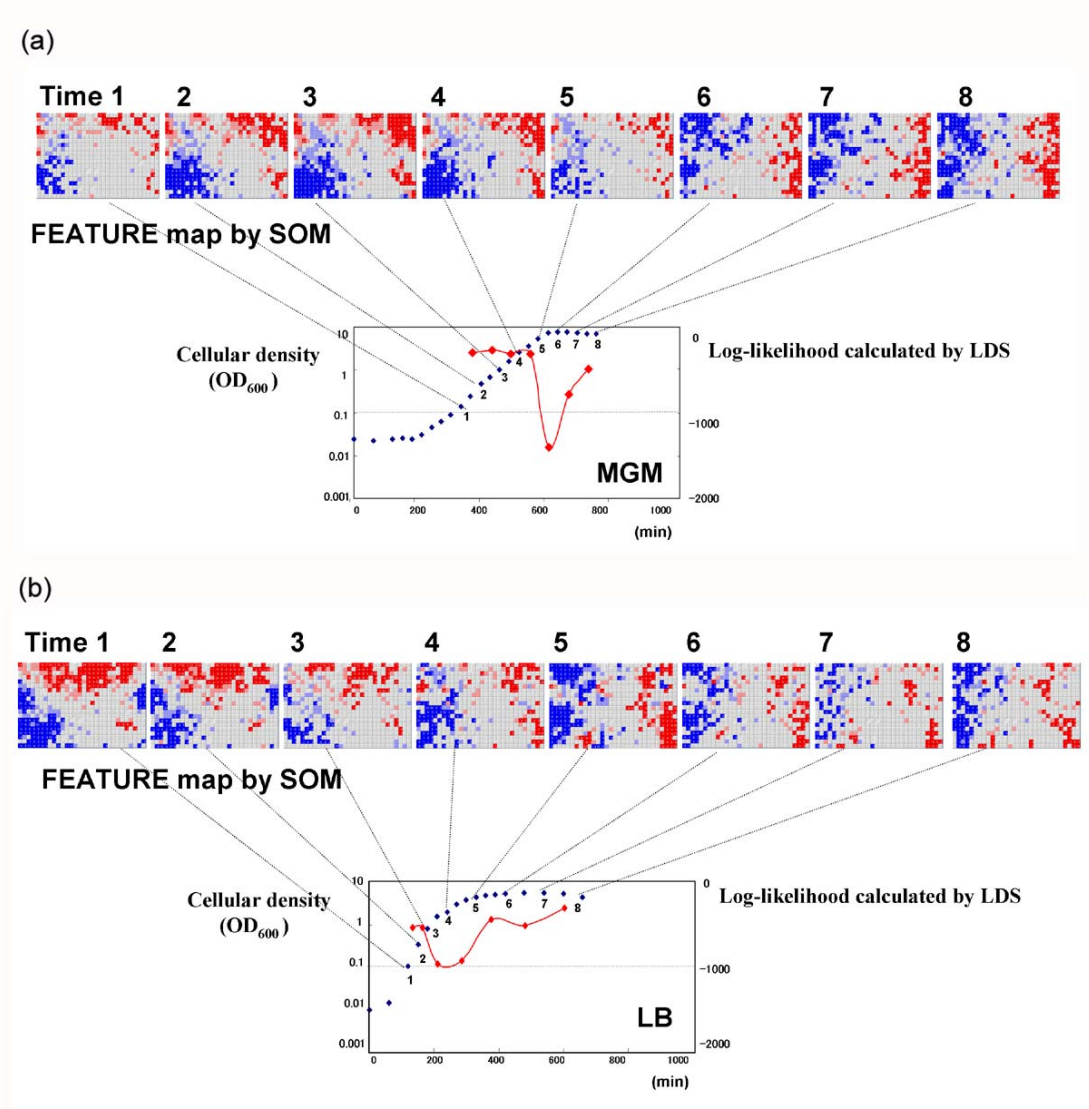
**Examples of the results of *Bacillus *data analyses**. The horizontal axis shows the culture time in minutes. The vertical axis represents the cellular density values (blue plots) and the probabilistic index for transition (red plots). a) The results of analysis of the LB data (control culture condition). According to the probabilistic index, the first transition was predicted between time points 3 and 4, the period that corresponded to transition from exponential growth phase to the stationary phase as indicated by cellular density values. The probabilistic index identifies another state change between time points 6 and 7 during the "stationary" phase. b) The results of analysis of the MGM data (stress culture condition). According to the probabilistic index, a state change was predicted between time points 5 and 6, a period that corresponded to the transition from the exponential growth phase to the stationary phase. Compared to the results from cells grown in LB, the transition timing was different. This difference was caused by the lack of glucose in MGM.

We first analyzed the relationship of cell population to state transition time on transcriptome data of *Bacillus subtilis *(Figure 1). Here, the exponential growth phase and stationary phase are commonly used microbiology terms referring to the state of the cellular population, as measured by the optical density (see also Methods section). The transition from exponential growth to the stationary phase was observed in 8 culture media: Lysogeny Broth (LB), Minimum Glucose Medium (MGM), Glucose Starvation (GS), Phosphate Starvation (PS), Competence Medium (CM), Difco Sporulation Medium (DSM), Competence Sporulation Medium (CSM) and DSM plus Glucose Glutamine (DGG). We confirmed that the log-likelihood index produced by LDS was smaller at the transition time between two phases. Next, we fitted the index calculated by the model to the phase transition data. For cell populations growing under two culture conditions, namely LB (control) and MGM (limited glucose), we found that BL-SOM yielded different classification results for gene expression (Figure 2a, b). This result indicates that expression of the genes responsible for the transition varied between the different environmental conditions, although their transitions appeared similar. For cells grown in either CSM or DSM, two transition points for sporulation were detected. The first was the well-known transition from exponential growth to the stationary phase. However, the second was a novel transition detected by this approach. At the first transition in CSM at around time point 3, log-likelihood values show a sustained drop. The analysis suggests that cells take a long time to adapt to the CSM culture environment. The second transition point in the sporulation media was further investigated by analysis of Feature maps generated by BL-SOM [7]. The candidate genes for the second transition were those activated just before the transition point and repressed soon after the transition point (Table 1). These genes are listed in Table 1 and include those related to lysis of the mother cell, such as *cwlH*. Thus, the second transition corresponded to mother cell lysis [9], a type of apoptosis.

**Table 1:** The list of transition driving genes identified in cells grown in CSM and DSM

**Functional categories**	**Genes**
Adaptation to typical conditions	*ypeB*
Cell wall	*ytcC, ykuG, ykoT, ywhE, yunA, cwlH*
Germination	*yaaH, yfkQ, yndD, gerBB, gerKB, yndE, gerAC, yfkR*
Membrane bioenergetics	*yhfW*
Sporulation	*spoVFA, spoVAD, spoVAC, spoVAE, spoVAB, spoVK, spoIVCA, cotC, cotA, yaaH, sspE, sspB*
Transport/binding proteins and lipoproteins	*ymfD, araP, yveA, ywcA, ywrK*
Detoxification	*ykoY*
Regulation	*sigG, splA*
Antibiotic production	*yitA, yitC*
Carbohydrates and related molecules	*yqiQ, adhB, yoaI, yesX, yitF, yqiQ*
Metabolism of amino acids and related molecules	*aprX, spoVFA*
Metabolism of lipids	*yngF*
Phage-related function	*yndL, yqbO, yqbQ*
Proteins with unknown function	*yodP, yheD, yhcQ, yvdQ, ytzC, yybC, ydfO, yhcV, yesV, yndM, ydfR, yngD, ykjA, yetA, yusN, yozN, yppD, ytlB, yqaN, ythQ, yycQ, yurS, yrkS, yxaG, yesJ, ysnE*

Using the analytical approach described here, we not only succeeded in detecting the well-known transition from exponential growth to the stationary phase, but also identified another, novel transition point. This result suggests the possibility that, even in periods that are assumed to be eventless, cells may be invoking their adaptive systems.

### Analysis of the *Arabidopsis *data

**Figure 3:**
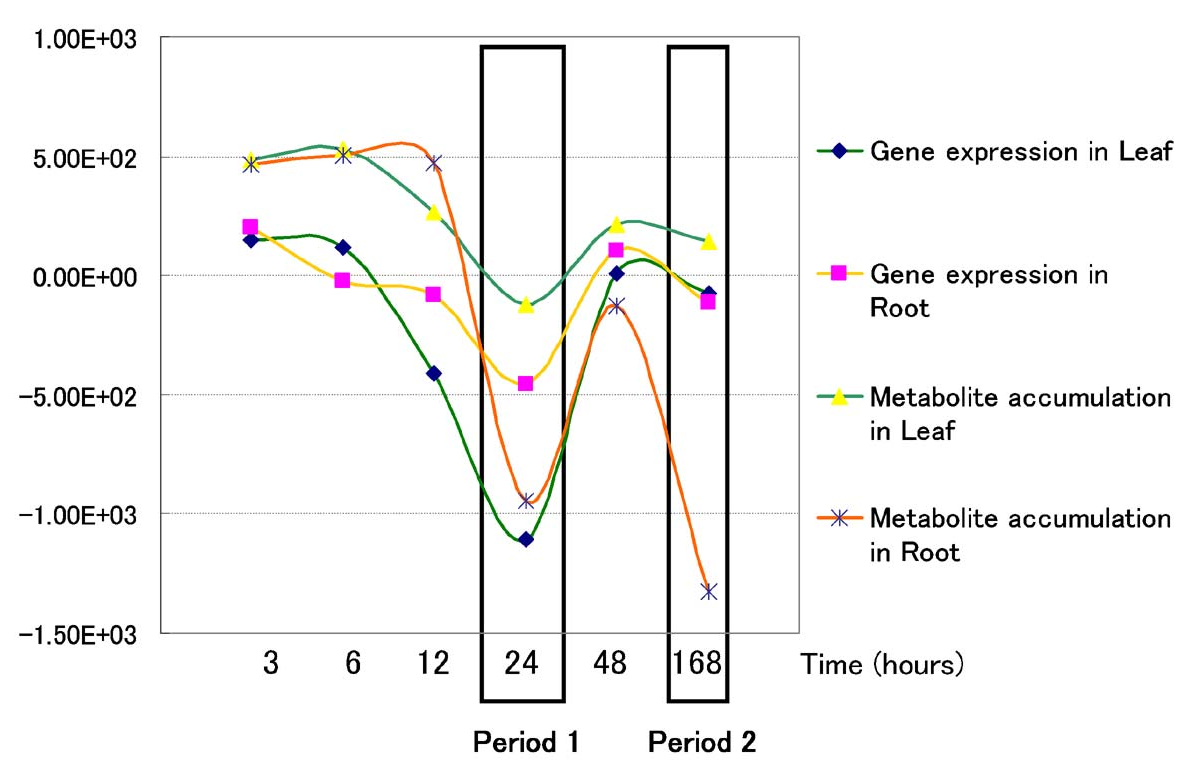
**The result of an LDS-based calculation showing a transition in gene expression and metabolite accumulation**. The ordinate scale indicates a log-likelihood value calculated by LDS. The transition in gene expression and metabolite accumulation in both leaf and root occurred most often in Period 1, showed by the left bold rectangle. During Period 2, shown by the right bold rectangle, a second transition in metabolite accumulation occurred solely in the root.

As described in the Methods section, we analysed changes in gene expression and metabolite accumulation in *Arabidopsis *plants following their transfer to sulfur-deficient conditions. We detected a transition between 12 and 24 hours in both gene expression and metabolite accumulation profiles in both leaves and roots. In addition, we detected a second transition at the final time point (168 hr) in the metabolite accumulation profile in roots (see Figure 3). At the transition point of 12–24 hr, glucosinolate biosynthesis was decreased in leaves and anthocyanin biosynthesis was initiated in roots. The predicted transitions obtained by this analysis are consistent with those identified previously [10], indicating that our method can reliably identify candidate genes and metabolites involved in transition points. The transition time point detected for root metabolites at the end of the experiment (168 hr) showed that even after this period of time roots of *A. thaliana *continued to change in response to sulfur deprivation, at least in terms of metabolite accumulation.

On the basis of the estimated transition results, coupled with prior knowledge and the Feature map subtraction obtained by BL-SOM, we identified metabolites whose accumulation profiles showed changes that coordinated with the predicted transition point. These metabolites were found to be involved in biochemical pathways that are critical for the response to sulfur deprivation stress, for example, glucosinolate biosynthesis in leaf and anthocyanin biosynthesis in root [10].

**Figure 4:**
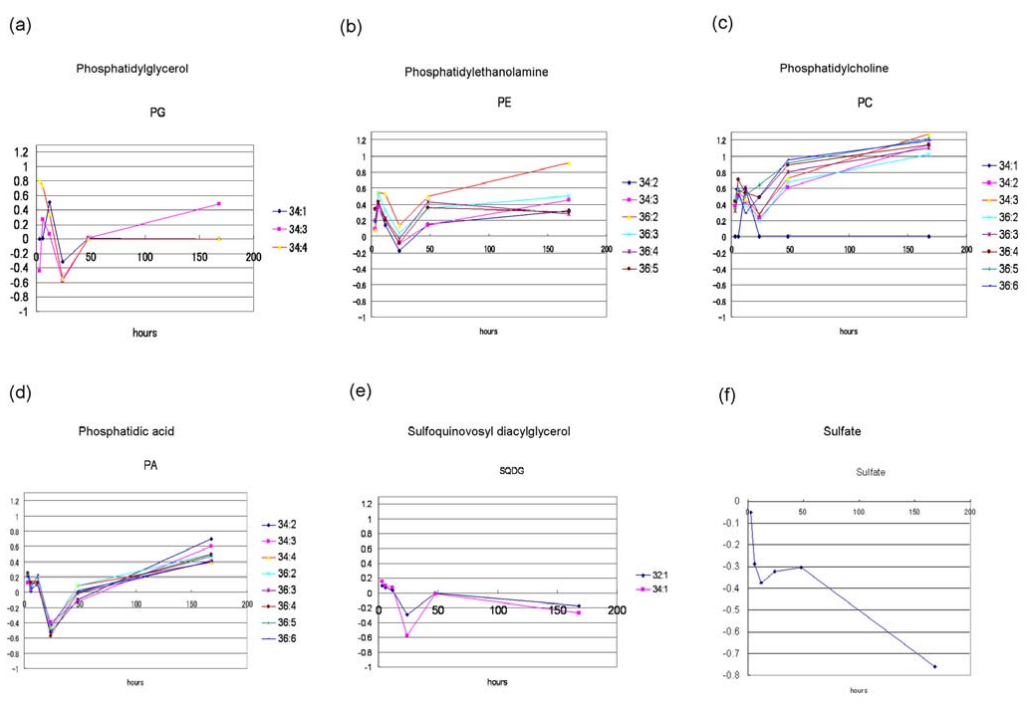
**Lipid accumulation profiles**. The accumulation profiles of metabolites whose m/z values corresponded to lipids expressing "total acyl carbon: total double bonds in two acyl groups", i.e., phosphatidylglycerol (a), phosphatidylethanolamine (b), phosphatidylcholine (c), phosphatidic acid (d), and sulfoquinovosyl diacylglycerol (e). the vertical axis shows normalized log-ratio values of the sulfur starvation condition to the control condition. (f) Sulfate profile analyzed by capillary electrophoresis. The vertical axis shows the log-ratio values of the sulfur starvation condition to the control condition.

Our results also suggested the presence of lipid metabolic responses in *Arabidopsis *to sulfur stress. The accumulation patterns of detected ion peaks whose mass-to-charge ratio (*m/z*) values corresponded to molecular species with various acyl groups, such as phosphatidylglycerol, phosphatidylethanolamine, phosphatidylcholine, phosphatidic acid, and sulfoquinovosyl diacylglycerol, are shown in Figure 4. Because the accumulation profiles of these compounds showed similar patterns, we predict that lipid biosynthesis was also co-ordinately repressed at the transition at 24 hr.

A profile of sulfate accumulation was generated using capillary electrophoresis (Figure 4f). In comparison with the control condition, the accumulation of sulfate was strongly repressed immediately after the shift to sulfur deficiency. Under sulfur deprivation, it was believed that sulfate levels (Figure 4f) would only decrease. During the transition period from 12 to 24 hr after the shift to sulfur deficiency, however, sulfate levels temporarily ceased declining and stayed relatively constant as compared to the control.

From these results, we hypothesize that sulfate is in an active form and is distributed throughout the plant at the transition time. During this period, in order to maintain the intracellular environment, membrane lipids are temporarily degraded and re-synthesized after the transition. This suggestion is consistent with the reported decrease in lipids under conditions of sulfur starvation [11].

## Conclusion

In summary, by using a linear dynamical system, we have identified transition times in the adaptation processes of *Bacillus subtilis *and *Arabidopsis thaliana *to environmental stresses. By focusing on transition information based on a well-defined probabilistic index, we obtained novel observations on apoptosis in *Bacillus subtilis *and the regulation of lipid metabolism connected with sulfur-stress responses in *Arabidopsis thaliana*. As this approach uses probabilistic values to detect the transitions, the results are objectively supported without the risk of misinterpretation due to experimental noise. The results of this approach will enable us to more effectively design experiments specifically tailored for functional identification of genes and metabolites. By obtaining time series data with higher temporal resolution around the transition time points, we can obtain more precise information on the details of the responses. The strategy described here was successful in identifying a small number of candidate genes and metabolites, from the vast number of genes and metabolites in comprehensive "omics" databases.

## Methods

### Time series data of *Bacillus subtilis*

The *Bacillus subtilis *time series data used in the present study were obtained from microarray analysis of cells sampled from 8 different experimental conditions. The data were produced using a two-colour fluorescence cDNA microarray that included 3100 *Bacillus subtilis *genes. The LB medium was developed to maximize cellular growth, and cells grown in this medium represented the control, unstressed population. In the initial phase of culture, the cell number increases by binary division – this is called the exponential growth phase in contrast to the stationary phase where the cell number has reached equilibrium. Data were collected from cells grown at 37°C in LB medium; the total length of culture was 12 hr and sampling was performed at 8 time points. Other culture conditions were also used with the aim of inducing stress responses in the cells. Cells were grown in Minimum Glucose Medium (MGM) at 37°C for 13 hr and sampled at 8 time points. Glucose starvation (GS) was achieved by eliminating the sugar from MGM; the cells were cultured in this medium for 10 hr and were sampled at 5 time points. Phosphate starvation (PS) was achieved by eliminating phosphoric acid from the MGM; the cells were cultured in this medium for 11 hr, and were sampled at 6 time points. Some cells were grown in Competence Medium (CM), which increases the ability of the cells to ingest DNA from the external environment. The cells were grown in CM for 9 hr and were sampled at 5 time points. Some cells were grown in Competence-Sporulation medium (CSM) for 15 hr and were sampled at 13 time points. A second sporulation medium, Difco sporulation medium (DSM), was also used. Cells were grown in this medium for 12 hr and were sampled at 19 time points. We also used Difco Glucose Glutamine (DGG) medium in which glucose and glutamine have been added to DSM medium in order to inhibit sporulation. The cells were grown for 9 hr in DSM and were sampled at 6 time points.

### Time series data of *Arabidopsis thaliana*

The *Arabidopsis thaliana *data used in the present study were obtained from DNA microarray experiments and by Fourier-transform ion cyclotron resonance mass spectrometry (FT-ICR-MS), as previously described [10]. In brief, *Arabidopsis *was cultured in sulfur-sufficient control medium for three weeks, transferred to control or sulfur-deprived medium, and cultured for up to one more week. Rosette leaves and roots were harvested at 3, 6, 12, 24, 48 and 168 hr after transfer, and subjected to transcriptome and metabolome analyses [10].

Transcriptome data were obtained using the Agilent Arabidopsis 2 microarray (Agilent Technologies, Palo Alto, CA), which carries 21,500 Arabidopsis genes [10]. The data are available on ArrayExpress in EMBL-EBI [12]. Non-targeted metabolome data were obtained by FT-ICR-MS [10], which produces precise mass-to-charge ratio values (m/z values) of metabolites [13]. Metabolites were provisionally identified from their m/z values and the analytical conditions used for FT-ICR-MS.

### Parameter estimation

The test distribution is defined as *q*(*X*_1:*T*_|*Y*_1:*T*_, *θ*) and is used to approximate the true posterior distribution. The Kullback-Leibler divergence takes the minimum value of 0 if the two distributions are equivalent.

In Equation (17), maximization of the free energy with respect to *q *and *θ *is equal to the calculation of the maximum likelihood estimate *θ *with respect to *Y*_1:*T*_.

F[qx,θ]≡log⁡p(Y1:T|θ)−KL[qx(X1:T)‖p(X1:T|Y1:T,θ)]=∫dX1:Tqx(X1:T)log⁡p(X1:T,Y1:T|θ)−∫dX1:Tqx(X1:T)log⁡qx(X1:T)
 MathType@MTEF@5@5@+=feaafiart1ev1aaatCvAUfKttLearuWrP9MDH5MBPbIqV92AaeXatLxBI9gBaebbnrfifHhDYfgasaacH8akY=wiFfYdH8Gipec8Eeeu0xXdbba9frFj0=OqFfea0dXdd9vqai=hGuQ8kuc9pgc9s8qqaq=dirpe0xb9q8qiLsFr0=vr0=vr0dc8meaabaqaciaacaGaaeqabaqabeGadaaakqaaeeqaaiabdAeagjabcUfaBjabdghaXnaaBaaaleaacqWG4baEaeqaaOGaeiilaWccciGae8hUdeNaeiyxa0LaeyyyIORagiiBaWMaei4Ba8Maei4zaCMaemiCaaNaeiikaGIaemywaK1aaSbaaSqaaiabigdaXiabcQda6iabdsfaubqabaGcdaabbaqaaiab=H7aXbGaay5bSdGaeiykaKIaeyOeI0Iaem4saSKaemitaWKaei4waSLaemyCae3aaSbaaSqaaiabdIha4bqabaGccqGGOaakcqWGybawdaWgaaWcbaGaeGymaeJaeiOoaOJaemivaqfabeaakiabcMcaPmaafeaabaGaemiCaaNaeiikaGIaemiwaG1aaSbaaSqaaiabigdaXiabcQda6iabdsfaubqabaGcdaabbaqaaiabdMfaznaaBaaaleaacqaIXaqmcqGG6aGocqWGubavaeqaaaGccaGLhWoacqGGSaalcqWF4oqCaiaawMa7aiabcMcaPiabc2faDbqaaiabg2da9maapeaabaGaemizaqMaemiwaG1aaSbaaSqaaiabigdaXiabcQda6iabdsfaubqabaGccqWGXbqCdaWgaaWcbaGaemiEaGhabeaakiabcIcaOiabdIfaynaaBaaaleaacqaIXaqmcqGG6aGocqWGubavaeqaaaqabeqaniabgUIiYdGccqGGPaqkcyGGSbaBcqGGVbWBcqGGNbWzcqWGWbaCcqGGOaakcqWGybawdaWgaaWcbaGaeGymaeJaeiOoaOJaemivaqfabeaakiabcYcaSiabdMfaznaaBaaaleaacqaIXaqmcqGG6aGocqWGubavaeqaaOWaaqqaaeaacqWF4oqCcqGGPaqkcqGHsisldaWdbaqaaiabdsgaKjabdIfaynaaBaaaleaacqaIXaqmcqGG6aGocqWGubavaeqaaOGaemyCae3aaSbaaSqaaiabdIha4bqabaGccqGGOaakcqWGybawdaWgaaWcbaGaeGymaeJaeiOoaOJaemivaqfabeaakiabcMcaPiGbcYgaSjabc+gaVjabcEgaNjabdghaXnaaBaaaleaacqWG4baEaeqaaOGaeiikaGIaemiwaG1aaSbaaSqaaiabigdaXiabcQda6iabdsfaubqabaGccqGGPaqkaSqabeqaniabgUIiYdaakiaawEa7aaaaaa@AE0D@

The free energy is maximized using the Expectation-Maximization algorithm [14] consisting of the following steps:

**Step 1**. Parameter set *θ *is initialized.

**Step 2**. E-step(step 2.1) and M-step (step 2.2) are successively repeated until the free energy converges.

**Step 2.1**. E-step:

*k *is a repeat loop index. By fixing the parameter *θ*^(*k*-1)^, *F *in Equation (17) is maximized with respect to *q*.

According to Equation (17), the solution is

qx(k)(X1:T)=p(X1:T|Y1:T,θ(k−1))=p(x1|Y1:T,θ(k−1)){∏t=2Tp(xt|xt−1,Y1:T,θ(k−1))}
 MathType@MTEF@5@5@+=feaafiart1ev1aaatCvAUfKttLearuWrP9MDH5MBPbIqV92AaeXatLxBI9gBamXvP5wqSXMqHnxAJn0BKvguHDwzZbqegyvzYrwyUfgarqqtubsr4rNCHbGeaGqiA8vkIkVAFgIELiFeLkFeLk=iY=Hhbbf9v8qqaqFr0xc9pk0xbba9q8WqFfeaY=biLkVcLq=JHqVepeea0=as0db9vqpepesP0xe9Fve9Fve9GapdbaqaaeGacaGaaiaabeqaamqadiabaaGcbaGaemyCae3aa0baaSqaaiabdIha4bqaaiabcIcaOiabdUgaRjabcMcaPaaakiabcIcaOiabdIfaynaaBaaaleaacqaIXaqmcqGG6aGocqWGubavaeqaaOGaeiykaKIaeyypa0JaemiCaaNaeiikaGIaemiwaG1aaSbaaSqaaiabigdaXiabcQda6iabdsfaubqabaGcdaabbaqaaiabdMfaznaaBaaaleaacqaIXaqmcqGG6aGocqWGubavaeqaaOGaeiilaWccciGae8hUdehacaGLhWoadaahaaWcbeqaaiabcIcaOiabdUgaRjabgkHiTiabigdaXiabcMcaPaaakiabcMcaPiabg2da9iabdchaWjabcIcaOiabhIha4naaBaaaleaacqaIXaqmaeqaaOWaaqqaaeaacqWGzbqwdaWgaaWcbaGaeGymaeJaeiOoaOJaemivaqLaeiilaWcabeaakiab=H7aXnaaCaaaleqabaGaeiikaGIaem4AaSMaeyOeI0IaeGymaeJaeiykaKcaaaGccaGLhWoacqGGPaqkdaGadaqaamaawahabeWcbaGaemiDaqNaeyypa0JaeGOmaidabaGaemivaqfaneaacqGHpis1aaGccqWGWbaCcqGGOaakcqWH4baEdaWgaaWcbaGaemiDaqhabeaakmaaeeaabaGaeCiEaG3aaSbaaSqaaiabdsha0jabgkHiTiabigdaXaqabaGccqGGSaalcqWGzbqwdaWgaaWcbaGaeGymaeJaeiOoaOJaemivaqfabeaakiabcYcaSiab=H7aXnaaCaaaleqabaGaeiikaGIaem4AaSMaeyOeI0IaeGymaeJaeiykaKcaaaGccaGLhWoacqGGPaqkaiaawUhacaGL9baaaaa@9787@

After the fixation of parameter *θ*, the calculations needed to calculate the value of Equation (18) are as follows:

When both the data *Y*_1:*t*-1 _and the parameter of the prior distribution *p*(*x*_*t*_|*Y*_1:*t*-1_, *θ*) of *x*_*t *_are given, the posterior distribution of *x*_*t*_, given the data *Y*_1:*t*_, is

p(xt|Y1:t,θ)=p(yt|xt,θ)p(xt|Y1:t−1,θ)∫dxtp(yt|xt,θ)p(xt|Y1:t−1,θ)
 MathType@MTEF@5@5@+=feaafiart1ev1aaatCvAUfKttLearuWrP9MDH5MBPbIqV92AaeXatLxBI9gBaebbnrfifHhDYfgasaacH8akY=wiFfYdH8Gipec8Eeeu0xXdbba9frFj0=OqFfea0dXdd9vqai=hGuQ8kuc9pgc9s8qqaq=dirpe0xb9q8qiLsFr0=vr0=vr0dc8meaabaqaciaacaGaaeqabaqabeGadaaakeaacqWGWbaCcqGGOaakcqWH4baEdaWgaaWcbaGaemiDaqhabeaakiabcYha8jabdMfaznaaBaaaleaacqaIXaqmcqGG6aGocqWG0baDaeqaaOGaeiilaWccciGae8hUdeNaeiykaKIaeyypa0ZaaSaaaeaacqWGWbaCcqGGOaakcqWH5bqEdaWgaaWcbaGaemiDaqhabeaakiabcYha8jabhIha4naaBaaaleaacqWG0baDaeqaaOGaeiilaWIae8hUdeNaeiykaKIaemiCaaNaeiikaGIaeCiEaG3aaSbaaSqaaiabdsha0bqabaGccqGG8baFcqWGzbqwdaWgaaWcbaGaeGymaeJaeiOoaOJaemiDaqNaeyOeI0IaeGymaedabeaakiabcYcaSiab=H7aXjabcMcaPaqaamaapeaabaGaemizaqMaeCiEaG3aaSbaaSqaaiabdsha0bqabaGccqWGWbaCcqGGOaakcqWH5bqEdaWgaaWcbaGaemiDaqhabeaakiabcYha8jabhIha4naaBaaaleaacqWG0baDaeqaaOGaeiilaWIae8hUdeNaeiykaKIaemiCaaNaeiikaGIaeCiEaG3aaSbaaSqaaiabdsha0bqabaGccqGG8baFcqWGzbqwdaWgaaWcbaGaeGymaeJaeiOoaOJaemiDaqNaeyOeI0IaeGymaedabeaakiabcYcaSiab=H7aXjabcMcaPaWcbeqab0Gaey4kIipaaaaaaa@803E@

Using (19), the prior distribution of *x*_*t*+1_, given the data *Y*_1:*t*_, is

*p*(*x*_*t*+1 _| *Y*_1:*t*_, *θ*) = ∫*d***x**_*t*_*p*(**x**_*t*+1 _| **x**_*t*_, *θ*)*p*(**x**_*t *_| *Y*_1:*T*_, *θ*)

By successively iterating Equations (19) and (20), *p*(**x**_*t *_| *Y*_1:*T *_*θ*) with arbitrary *t *is obtained. This repeating method is called the Kalman Filter [8].

If all data are given, the following joint probability is obtained:

p(xt+1,xt|Y1:T,θ)=p(xt+1|Y1:T,θ)p(xt+1|xt,θ)p(xt|Y1:t,θ)∫dxtp(xt+1|xt,θ)p(xt|Y1:t,θ)
 MathType@MTEF@5@5@+=feaafiart1ev1aaatCvAUfKttLearuWrP9MDH5MBPbIqV92AaeXatLxBI9gBaebbnrfifHhDYfgasaacH8akY=wiFfYdH8Gipec8Eeeu0xXdbba9frFj0=OqFfea0dXdd9vqai=hGuQ8kuc9pgc9s8qqaq=dirpe0xb9q8qiLsFr0=vr0=vr0dc8meaabaqaciaacaGaaeqabaqabeGadaaakeaacqWGWbaCcqGGOaakcqWH4baEdaWgaaWcbaGaemiDaqNaey4kaSIaeGymaedabeaakiabcYcaSiabhIha4naaBaaaleaacqWG0baDaeqaaOWaaqqaaeaacqWGzbqwdaWgaaWcbaGaeGymaeJaeiOoaOJaemivaqfabeaakiabcYcaSGGaciab=H7aXbGaay5bSdGaeiykaKIaeyypa0JaemiCaaNaeiikaGIaeCiEaG3aaSbaaSqaaiabdsha0jabgUcaRiabigdaXaqabaGcdaabbaqaaiabdMfaznaaBaaaleaacqaIXaqmcqGG6aGocqWGubavaeqaaOGaeiilaWIae8hUdehacaGLhWoacqGGPaqkdaWcaaqaaiabdchaWjabcIcaOiabhIha4naaBaaaleaacqWG0baDcqGHRaWkcqaIXaqmaeqaaOWaaqqaaeaacqWH4baEdaWgaaWcbaGaemiDaqhabeaakiabcYcaSiab=H7aXjabcMcaPiabdchaWjabcIcaOiabhIha4naaBaaaleaacqWG0baDaeqaaOWaaqqaaeaacqWGzbqwdaWgaaWcbaGaeGymaeJaeiOoaOJaemiDaqhabeaakiabcYcaSiab=H7aXjabcMcaPaGaay5bSdaacaGLhWoaaeaadaWdbaqaaiabdsgaKjabhIha4naaBaaaleaacqWG0baDaeqaaOGaemiCaaNaeiikaGIaeCiEaG3aaSbaaSqaaiabdsha0jabgUcaRiabigdaXaqabaGcdaabbaqaaiabhIha4naaBaaaleaacqWG0baDaeqaaOGaeiilaWIae8hUdeNaeiykaKIaemiCaaNaeiikaGIaeCiEaG3aaSbaaSqaaiabdsha0bqabaGcdaabbaqaaiabdMfaznaaBaaaleaacqaIXaqmcqGG6aGocqWG0baDaeqaaOGaeiilaWIae8hUdeNaeiykaKcacaGLhWoaaiaawEa7aaWcbeqab0Gaey4kIipaaaaaaa@96E2@

If the parameter of *P*(*x*_*t*+1_, | *Y*_1:*T*_, *θ*) is given, the following distribution is obtained:

*p*(**x**_*t *_| *Y*_1:*T*_, *θ*) = ∫*d***x**_*t*+1_*p*(**x**_*t*+1_, **x**_*t *_| *Y*_1:*T*_, *θ*)

By successively iterating Equations (21) and (22), *p*(**x**_*t*+1_, **x**_*t*_|*Y*_1:*T*_, *θ*) and *p*(**x**_*t*_|*Y*_1:*T*_, *θ*), which are necessary to calculate the value of Equation (18), are obtained.

This repeating method is called the Kalman smoother [15].

Using the Kalman smoother, the statistical values necessary for parameter estimation are obtained.

If *p*(**x**_*t*_|*Y*_1:*t*-1_, *θ*) is given, the following likelihood is calculated:

*p*(**y**_*t*_|*Y*_1:*t*-1_, *θ*) = ∫ *d***x**_*t*_*p*(**y**_*t*_,|**x**_*t*_, *θ*)*p*(**x**_*t*_|*Y*_1:*t*-1_, *θ*)

Using (23), the log-likelihood is calculated as

log⁡p(Y1:T|θ)=∑t=1Tlog⁡p(yt|Y1:t−1,θ)
 MathType@MTEF@5@5@+=feaafiart1ev1aaatCvAUfKttLearuWrP9MDH5MBPbIqV92AaeXatLxBI9gBaebbnrfifHhDYfgasaacH8akY=wiFfYdH8Gipec8Eeeu0xXdbba9frFj0=OqFfea0dXdd9vqai=hGuQ8kuc9pgc9s8qqaq=dirpe0xb9q8qiLsFr0=vr0=vr0dc8meaabaqaciaacaGaaeqabaqabeGadaaakeaacyGGSbaBcqGGVbWBcqGGNbWzcqWGWbaCcqGGOaakcqWGzbqwdaWgaaWcbaGaeGymaeJaeiOoaOJaemivaqfabeaakiabcYha8HGaciab=H7aXjabcMcaPiabg2da9maaqahabaGagiiBaWMaei4Ba8Maei4zaCMaemiCaaNaeiikaGIaeCyEaK3aaSbaaSqaaiabdsha0bqabaGccqGG8baFcqWGzbqwdaWgaaWcbaGaeGymaeJaeiOoaOJaemiDaqNaeyOeI0IaeGymaedabeaakiabcYcaSiab=H7aXjabcMcaPaWcbaGaemiDaqNaeyypa0JaeGymaedabaGaemivaqfaniabggHiLdaaaa@58AB@

**Step 2.2**. M-step

In this step, the value of *θ *that will maximize *F *under the condition *q*_*x *_= *q*_*x*_^(*k*) ^is calculated using Equation (25):

θ(k)=max⁡θ{∫dX1:Tqx(k)(X1:T)log⁡p(X1:T,Y1:T|θ)}
 MathType@MTEF@5@5@+=feaafiart1ev1aaatCvAUfKttLearuWrP9MDH5MBPbIqV92AaeXatLxBI9gBamXvP5wqSXMqHnxAJn0BKvguHDwzZbqegyvzYrwyUfgarqqtubsr4rNCHbGeaGqiA8vkIkVAFgIELiFeLkFeLk=iY=Hhbbf9v8qqaqFr0xc9pk0xbba9q8WqFfeaY=biLkVcLq=JHqVepeea0=as0db9vqpepesP0xe9Fve9Fve9GapdbaqaaeGacaGaaiaabeqaamqadiabaaGcbaacciGae8hUde3aaWbaaSqabeaacqGGOaakcqWGRbWAcqGGPaqkaaGccqGH9aqpdaWfqaqaaiGbc2gaTjabcggaHjabcIha4bWcbaGae8hUdehabeaakmaacmaabaWaa8qaaeaacqWGKbazcqWGybawdaWgaaWcbaGaeGymaeJaeiOoaOJaemivaqfabeaakiabdghaXnaaDaaaleaacqqG4baEaeaacqqGOaakcqqGRbWAcqqGPaqkaaGccqGGOaakcqWGybawdaWgaaWcbaGaeGymaeJaeiOoaOJaemivaqfabeaakiabcMcaPiGbcYgaSjabc+gaVjabcEgaNjabdchaWjabcIcaOiabdIfaynaaBaaaleaacqaIXaqmcqGG6aGocqWGubavaeqaaOGaeiilaWIaemywaK1aaSbaaSqaaiabigdaXiabcQda6iabdsfaubqabaGcdaabbaqaaiab=H7aXjabcMcaPaGaay5bSdaaleqabeqdcqGHRiI8aaGccaGL7bGaayzFaaaaaa@73FF@

The objective function to be maximized is defined as

*J*(*θ*) = ∫*dX*_1:*T*_*q*_*x*_^(*k*)^(*X*_1:*T*_)log *p*(*X*_1:*T*_, *Y*_1:*T*_|*θ*)

which is obtained by the following equation:

∂J(θ)∂θ=0
 MathType@MTEF@5@5@+=feaafiart1ev1aaatCvAUfKttLearuWrP9MDH5MBPbIqV92AaeXatLxBI9gBaebbnrfifHhDYfgasaacH8akY=wiFfYdH8Gipec8Eeeu0xXdbba9frFj0=OqFfea0dXdd9vqai=hGuQ8kuc9pgc9s8qqaq=dirpe0xb9q8qiLsFr0=vr0=vr0dc8meaabaqaciaacaGaaeqabaqabeGadaaakeaadaWcaaqaaiabgkGi2kabdQeakjabcIcaOGGaciab=H7aXjabcMcaPaqaaiabgkGi2kab=H7aXbaacqGH9aqpcqaIWaamaaa@37B9@

and the solution of parameter *θ *is calculated that maximizes *F*.

Parameter *θ *is then updated, and the process goes back to E-step.

## Competing interests

The author(s) declares that there are no competing interests.

## Authors' contributions

RM designed the LDS method and carried out the computer simulations. SK designed the BL-SOM and carried out the computer experiments. MYH  supplied the Arabidopsis dataset. MY analyzed FT-MS data with RM. NO supervised the *Bacillus *experiments. KS proposed and supervised the study. All authors read and approved the final manuscript.
